# Changes in virus detection in hospitalized children before and after the severe acute respiratory syndrome coronavirus 2 pandemic

**DOI:** 10.1111/irv.12995

**Published:** 2022-04-29

**Authors:** Yohei Kume, Koichi Hashimoto, Mina Chishiki, Sakurako Norito, Reiko Suwa, Takashi Ono, Izumi Mochizuki, Fumi Mashiyama, Naohisa Ishibashi, Shigeo Suzuki, Hiroko Sakuma, Hitoshi Takahashi, Makoto Takeda, Kazuya Shirato, Mitsuaki Hosoya

**Affiliations:** ^1^ Department of Pediatrics Fukushima Medical University Fukushima Japan; ^2^ Department of Virology III National Institute of Infectious Diseases Musashimurayama Japan; ^3^ Department of Pediatrics Ohara General Hospital Fukushima Japan; ^4^ Department of Pediatrics Hoshi General Hospital Koriyama Japan; ^5^ Influenza and Respiratory Virus Research Center National Institute of Infectious Diseases Musashimurayama Japan

**Keywords:** children, epidemiology, hospitalization, human metapneumovirus, influenza virus, SARS‐CoV‐2

## Abstract

The impact of strengthening preventive measures against severe acute respiratory syndrome coronavirus 2 (SARS‐CoV‐2) infection on the prevalence of respiratory viruses in children was examined. After the SARS‐CoV‐2 pandemic, the rate of multiple virus detection among hospitalized children decreased. Immediately after the SARS‐CoV‐2 pandemic, respiratory syncytial and parainfluenza viruses were rarely detected and subsequently reemerged. Human metapneumovirus and influenza virus were not consistently detected. Non‐enveloped viruses (bocavirus, rhinovirus, and adenovirus) were detected to some extent even after the pandemic. Epidemic‐suppressed infectious diseases may reemerge as susceptibility accumulates in the population and should continue to be monitored.

## 1 INTRODUCTION

### 1.1 Background

Respiratory tract infections are one of the most common reasons for hospitalization in children. Respiratory viral infections are mainly transmitted through human‐to‐human contact. After the severe acute respiratory syndrome coronavirus 2 (SARS‐CoV‐2) pandemic, contact and movement of people were restricted to prevent the spread of SARS‐CoV‐2 in Japan. Thorough measures to prevent infection with SARS‐CoV‐2 have significantly altered the prevalence of other respiratory virus infections in children.^1^, ^2^, ^3^, ^4^ However, changes in the proportion of respiratory infection viruses detected in children hospitalized before and after the SARS‐CoV‐2 pandemic in Japan remain unknown.

To clarify changes in the epidemic dynamics of respiratory infection due to the impact of thorough infection prevention measures during the SARS‐CoV‐2 pandemic, we examined changes in the detection rate of respiratory viruses in specimens collected from all children with respiratory infections admitted to single facility from January 2018 to December 2021.

## 2 MATERIALS AND METHODS

### 2.1 Study design

This study has been carried out in accordance with the Declaration of Helsinki. In this observational study, nasopharyngeal swabs were collected from all children under the age of 15 years who had respiratory symptoms on admission at a single secondary medical center in the Fukushima Prefecture between January 2018 and December 2021. Informed consent was obtained from all parents. The swabs were cryopreserved at −80°C until further use. The following 18 respiratory viruses were detected by real‐time reverse transcription polymerase chain reaction (RT‐PCR): respiratory syncytial virus (RSV) A and B; influenza virus (Flu) A, B, and C; human coronavirus (HCoV) 229E, HKU1, NL63, and OC43; human metapneumovirus (HMPV); human parainfluenza (HPIV) 1, 2, 3, and 4; human rhinovirus (HRV); adenovirus (HAdV) 2 and 4; and human bocavirus (HBoV). Each primer, probe, and reaction conditions were set based on previous reports^5^, ^6^, ^7^, ^8^, ^9^, ^10^, ^11^, ^12^ and Table S1. Viral nucleic acids were extracted using the QIAamp viral RNA mini kit (Qiagen, Hilden, Germany), QIAamp 96 Virus QIAcube HT kit, or Nucleospin 96 Virus (Macherey‐Nagel, Düren, Germany). One‐step RT‐PCR was performed using AgPath‐ID ™ One‐Step RT‐PCR (Thermo Fisher Scientific, Waltham, MA, USA) reagents. In two‐step RT‐PCR, cDNA was synthesized with random hexamer primers and Oligo (dT)12‐18 Primer (Thermo Fisher Scientific) using SMART M‐MLV Reverse Transcriptase (Takara Bio, Shiga, Japan). The LightCycler 480 Probes Master (Roche, Basel, Switzerland) was used for a two‐step RT‐PCR.

Data on age at admission, sex, airway symptoms, and diagnosis were obtained from medical records. Children infected with SARS‐CoV‐2 and those who had contact with SARS‐CoV‐2‐infected people were excluded from this study.

In Japan, the first SARS‐CoV‐2 case was confirmed in January 2020,^13^ followed by the start of the pandemic; the first emergency declaration was issued in April 2020 to control the SARS‐CoV‐2 pandemic.

## 3 RESULTS

**FIGURE 1:**
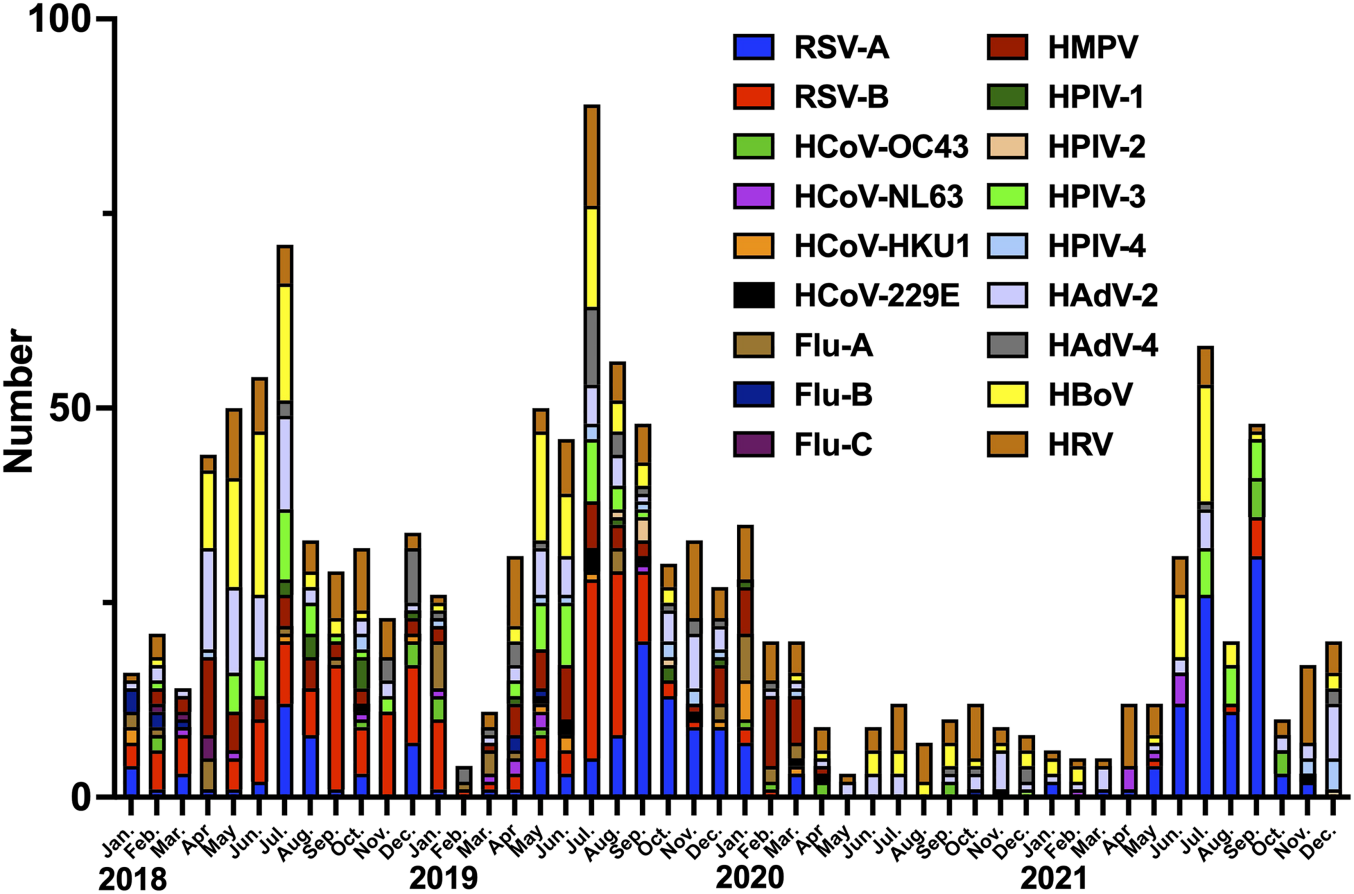
The change in the total number of viruses detected by polymerase chain reaction (PCR) in the present study from the 4‐year survey period. Flu, influenza virus; HAdV, human adenovirus; HBoV, human bocavirus; HCoV, human coronavirus; HMPV, human metapneumovirus; HPIV, human parainfluenza virus; HRV, human rhinovirus; RSV, respiratory syncytial virus

**FIGURE 2:**
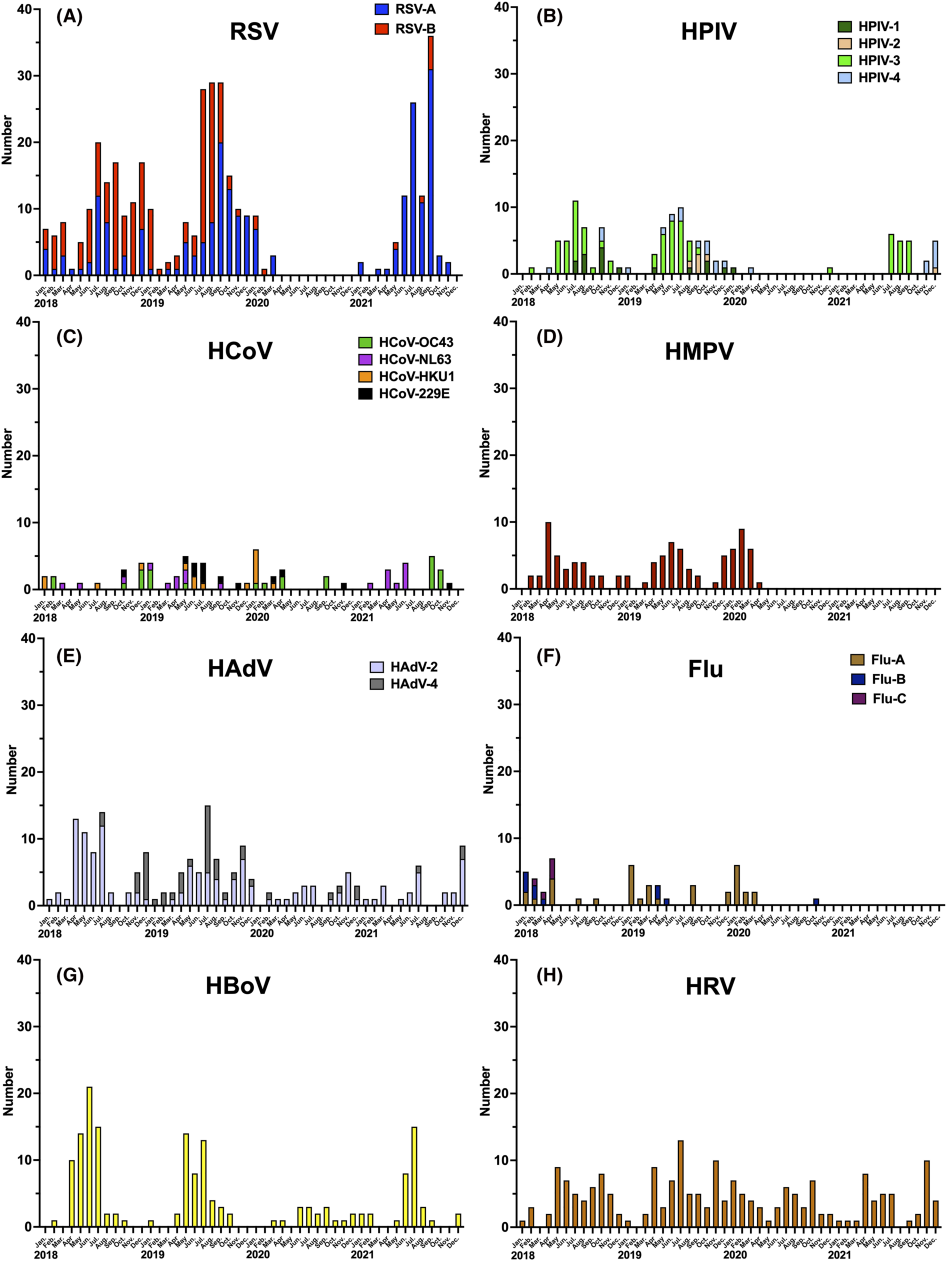
The number of patients with each virus per month during the 4‐year survey period. (A) RSV A and B; (B) HPIV 1, 2, 3, and 4; (C) HCoV‐HKU1, OC43, NL63, and 229E; (D) HMPV; (E) HAdV 2 and 4; (F) Flu A, B, and C; (G) HBoV; (H) HRV. Flu, influenza virus; HAdV, human adenovirus; HBoV, human bocavirus; HCoV, human coronavirus; HMPV, human metapneumovirus; HPIV, human parainfluenza virus; HRV, human rhinovirus; RSV, respiratory syncytial virus

During the 4‐year study period, 1165 children were hospitalized with symptoms of respiratory tract infections. The median age of admitted children was 18 months (interquartile range 10–37 months), with a higher proportion of them being boys (53.6%, 626/1165). Of the 1165 hospitalized children, viruses were detected in 877 (75.3%): 563 (48.3%) with a single virus and 314 (27.0%) with multiple viruses. RSV (388/1247, 33.3%) was the most frequently detected virus in this study (Table 1). The year with the lowest number of hospitalized children presenting with symptoms of respiratory tract infections was 2020, with 14.9 patients/month, which was less than half of the highest number of hospitalized patients in 2019 (32.1 patients/month). Furthermore, the virus detection rate (66.5%, 119/179) and virus duplication detection rate (16.2%, 29/179) in 2020, when the SARS‐CoV‐2 pandemic began, were also lower than those recorded in other years. Figure 1 shows the change in the total number of viruses detected by PCR in the present study from 2018 to 2021. If the multiple viruses were detected in a single specimen, each virus was counted. The overall number of viruses detected decreased significantly after April 2020, when a state of emergency was first declared in Japan, and then the number of viruses detected increased again from June 2020. Figure 2 shows the changes in each viral epidemic dynamics detected from January 2018 to December 2021. Since April 2020, the number of viruses detected has decreased significantly. In particular, RSV, HPIV, HMPV, and Flu were almost undetectable from April 2020 to April 2021; RSV and HPIV reemerged in the summer of 2021 and the number of hospitalizations increased, but HMPV and Flu were almost undetectable until December 2021. Meanwhile, a certain number of HRV, HAdV, and HBoV cases were detected from April 2020 to April 2021, although the number of detected cases decreased.

**TABLE 1:** Clinical characteristics

Duration	2018–2021 (*N* = 1165)	2018 (*n* = 358)	2019 (*n* = 385)	2020 (*n* = 179)	2021 (*n* = 243)
	Median [IQR]/Number (%)	Median [IQR]/Number (%)	Median [IQR]/Number (%)	Median [IQR]/Number (%)	Median [IQR]/Number (%)
The number of hospitalizations per month (/month)	24.3^a^	29.8^a^	32.1^a^	14.9^a^	20.3^a^
Age (months)	18 [10–37]	16 [8–30]	19 [10–41]	19 [12–46]	19 [10–31]
Sex (male)	626 (53.7)	190 (53.1)	218 (56.6)	96 (53.6)	122 (50.2)
Virus detection rate	877 (75.3)	276 (77.1)	296 (76.9)	119 (66.5)	186 (76.5)
Single virus detection	563 (48.3)	160 (44.6)	179 (46.5)	90 (50.3)	134 (55.1)
Multiple virus detection	314 (27.0)	116 (32.4)	117 (30.4)	29 (16.2)	52 (21.4)
RSV (A and B)	388 (33.3)	125 (34.9)	150 (39.0)	13 (7.3)	100 (41.2)
Flu (A, B, C)	50 (4.3)	20 (5.6)	19 (4.9)	11 (6.1)	0 (0.0)
HCoV (HKU1, OC43, 229E, and NL63)	67 (5.8)	11 (3.1)	24 (6.2)	15 (8.4)	17 (7.0)
HMPV	94 (8.1)	36 (10.1)	36 (9.4)	22 (12.3)	0 (0.0)
HPIV (1, 2, 3, 4)	110 (9.4)	39 (10.9)	47 (12.2)	3 (1.7)	21 (8.6)
HAdV (2, 4)	178 (15.3)	67 (18.7)	62 (16.1)	24 (14.3)	25 (10.3)
HBoV	164 (14.1)	66 (18.4)	47 (12.2)	17 (9.5)	34 (14.0)
HRV	205 (17.6)	52 (14.5)	62 (16.1)	48 (26.8)	43 (17.7)

*Note*: Data are shown as median (IQR) or numbers (%).

Abbreviations: Flu, influenza virus; HAdV, human adenovirus; HBoV, human bocavirus; HCoV, human coronavirus; HMPV, human metapneumovirus; HPIV, human parainfluenza virus; HRV, human rhinovirus; IQR, interquartile range; RSV, respiratory syncytial virus.

^a^ The number of children hospitalized with respiratory symptoms per month.

## 4 DISCUSSION

Confirming changes in the epidemic dynamics of the non‐SARS‐CoV‐2 respiratory viruses before and after the SARS‐CoV‐2 pandemic helps reveal the effects of infection control by suppressing contact with people and the changes in epidemic dynamics caused by the deterioration in herd immunity. The present study showed that the number of children hospitalized for symptoms of respiratory tract decreased, and the epidemic dynamics of respiratory viruses changed after the SARS‐CoV‐2 pandemic. RSV and HPIV had a resurgence after a non‐epidemic period, whereas HMPV and Flu have not been consistently detected since April 2020. Furthermore, this study revealed that multiple virus detection rates decreased after the SARS‐CoV‐2 pandemic, compared with those before the pandemic.

The first state of emergency was declared in April 2020 to control the spread of the SARS‐CoV‐2 pandemic, and the daily lifestyle in Japan changed. Specifically, due to school closures and recommendations for working at home, contact between people has decreased significantly. Even though kindergartens and nurseries were not closed, the number of pediatric outpatient visits decreased significantly.^3^ In addition, thorough infection prevention measures such as ventilation and alcohol disinfection, as well as changes in behavioral awareness, such as refraining from attending nursery schools and kindergartens when mild cold symptoms appear, may have reduced opportunities for children to become infected and contributed to the reduction of respiratory viral infections other than SARS‐CoV‐2 and multiple virus detection. Furthermore, a study reported that thorough measures to prevent infection suppressed not only respiratory infections but also a gastroenteritis epidemic.^1^

Although the number of detected HRV, HAdV, and HBoV cases decreased after thorough measures to prevent infection with SARS‐CoV‐2, these viruses were consistently detected even after the SARS‐CoV‐2 pandemic.

Previous reports have shown an increased risk of HRV during the SARS‐CoV‐2 pandemic.^4^ This may be due to the relatively low sensitivity of non‐envelopes to alcohol, which may have resulted in inadequate protection against non‐enveloped viruses.^4^ Furthermore, previous studies have shown that masks are less protective against HRV infection than against Flu or HCoV.^14^ Nevertheless, our results suggest that standard prophylaxis against SARS‐CoV‐2 does not eliminate not only HRV but also non‐enveloped viruses such as HBoV and HAdV.

After the SARS‐CoV‐2 pandemic, the risk of respiratory virus infection decreased in 2020, but RSV and HPIV reemerged in the summer of 2021. The reemergence of these viruses may be attributed to behavioral moderation in the community and the increase in susceptibility among the population.^15^ Meanwhile, HMPV and Flu did not reemerge by December 2021 during the study period. Population immunity to HMPV and influenza virus is also likely to have declined, and we need to pay attention to the re‐emergence of these viruses in Japan eventually.

A limitation of this study is that it was conducted at a single institution. However, the facility from which the specimens were collected was the core pediatric hospital in the area. Therefore, it is considered to strongly reflect the prevalence of pediatric infectious diseases in that region.

In conclusion, after the SARS‐CoV‐2 pandemic, the number of children hospitalized for respiratory virus infection and multiple detection of viruses decreased significantly, and subsequently, some viruses, including RSV and HPIV, had a resurgence. In contrast, a certain number of HRV, HAdV, and HBoV, which are classified as non‐enveloped viruses, were detected during the SARS‐CoV‐2 pandemic. Thorough precautions against SARS‐CoV‐2 significantly changed the status of herd immunity for non‐SARS‐CoV‐2 respiratory virus infections in children. HMPV and Flu, which were not prevalent during the study period, may become widespread. Therefore, continued epidemiological investigations of respiratory virus infections in the future are required.

## AUTHOR CONTRIBUTIONS

**Yohei Kume**: Conceptualization (equal), data curation (equal), formal analysis (lead), original draft preparation (lead); **Koichi Hashimot**o: Supervision (equal), review and editing (lead), conceptualization (equal): **Mina Chishiki**: Investigation (equal); **Sakurako Norito**: Investigation (equal), data curation (equal); **Reiko Suwa**: Investigation (equal); **Takashi Ono**: Investigation (equal); **Izumi Mochizuki**: Investigation (equal); **Fumi Mashiyama**: Investigation (equal); **Naohisa Ishibashi**: Investigation (equal); **Shigeo Suzuki**: Investigation (equal); **Hiroko Sakuma**: Investigation (equal); **Hitoshi Takahashi**, Methodology (equal); **Makoto Takeda**: Supervision (equal); **Kazuya Shirato**: Supervision (equal), methodology (equal); **Mitsuaki Hosoya**: Supervision (equal).

### PEER REVIEW

The peer review history for this article is available at https://publons.com/publon/10.1111/irv.12995.

## Supporting information


Supporting Information S1

## Data Availability

The data that support the findings of this study are available from the corresponding author upon reasonable request.
