# Supplementary material for: Changes in virus detection in hospitalized children before and after the severe acute respiratory syndrome coronavirus 2 pandemic
Source: Influenza Other Respir Viruses. 2022 Apr 29;16(5):837–41. doi: 10.1111/irv.12995 (PMC9343337; doi:10.1111/irv.12995)
Supplement: Supplementary file 1 — Supporting Information S1 [file IRV-16-837-s001.docx]

**Supplement table** Primers and probes for HCoV-HKU1

| Pathogen  (target) | Primer and Probe | Sequence, (5'-3') | length |
| --- | --- | --- | --- |
| HCoV-HKU1  (Nucleocapsid gene) | HKU1_N-F  HKU1_N-R  HKU1_N-Probe | GTTGCTAATCACCAAGCTGACAC  CGTACCAGGCGGAAACCTAG  (FAM) CCCTCCGATGTTTCGTCAAGGGATCCT (BHQ1) | 23  20  27 |

HCoV, human coronavirus; FAM, fluorescein amidite; BHQ, Black hole quencher
